# Isolation leads to greater clonality and reduced seed production in a temperate seagrass

**DOI:** 10.1093/aob/mcag008

**Published:** 2026-01-23

**Authors:** Timothy M Smith, Georgina Bramwell, Eric A Treml, Paul H York, Peter I Macreadie, D Jeff Ross, Michael J Keough, Craig D H Sherman

**Affiliations:** School of Life and Environmental Science, Deakin University Queenscliff Marine Science Centre, Pigdons Road, Waurn Ponds, VIC 3217, Australia; Centre for Tropical Water & Aquatic Ecosystem Research (TropWATER), James Cook University, PO Box 6811, Cairns, QLD 4870, Australia; School of Life and Environmental Science, Deakin University Queenscliff Marine Science Centre, Pigdons Road, Waurn Ponds, VIC 3217, Australia; School of Life and Environmental Science, Deakin University Queenscliff Marine Science Centre, Pigdons Road, Waurn Ponds, VIC 3217, Australia; School of BioSciences, University of Melbourne, Melbourne, Victoria 3010, Australia; Centre for Tropical Water & Aquatic Ecosystem Research (TropWATER), James Cook University, PO Box 6811, Cairns, QLD 4870, Australia; School of Life and Environmental Science, Deakin University Queenscliff Marine Science Centre, Pigdons Road, Waurn Ponds, VIC 3217, Australia; Centre for Nature Positive Solutions, Biosciences and Food Technology Discipline, School of Science, RMIT University, Melbourne, VIC 3000, Australia; Institute for Marine and Antarctic Studies, University of Tasmania, Taroona, TAS 7053, Australia; School of BioSciences, University of Melbourne, Melbourne, Victoria 3010, Australia; School of Life and Environmental Science, Deakin University Queenscliff Marine Science Centre, Pigdons Road, Waurn Ponds, VIC 3217, Australia

**Keywords:** Clones, hydrodynamics, genotypic richness, reproductive strategies, modular organisms, *Heterozostera nigricaulis*, seagrass

## Abstract

**Background and Aim:**

Many plants have complex mating systems involving sexual and asexual reproduction. Investment in different reproductive strategies can vary among sub-populations and is linked to local ecological conditions, but the key drivers are not well understood. We aimed to use direct estimates of reproductive investment (flowering and seed production), population genetic surveys and a biophysical model to assess the relationship between connectivity and the relative importance of sexual and asexual reproduction in maintaining seagrass populations. We predicted that populations with high levels of connectivity and investment in flowering and seed production will display higher levels of genotypic diversity, while more isolated populations with lower investment in flowering and seed production will display higher levels of clonality.

**Methods:**

We combined field surveys of flowering and seed production with population genetic surveys and a biophysical dispersal model to assess reproductive effort and patterns of connectivity in the seagrass *Heterozostera nigricaulis* across 16 sites in a large embayment in south-eastern Australia.

**Key Results:**

Estimates of genotypic diversity varied widely between locations, ranging from highly clonal (*R* = 0.18) to highly diverse (*R* = 0.91). Genotypic diversity correlated strongly with local seed production and the inflow of propagules derived from the biophysical dispersal model (pseudo-*R*^2^ = 0.73). Sites that receive low numbers of propagules and produce few seeds were more clonal than sites with high propagule inflow and seed density.

**Conclusions:**

These results show that isolated populations have higher levels of clonality and invest less in sexual reproduction. This has important consequences for the managing of declining populations of seagrass where fragmentation and loss of key source populations of propagules may lead to declines in genotypic and genetic diversity and the long-term viability of these important habitat-forming species.

## INTRODUCTION

Many organisms balance resource allocation between sexual and asexual reproduction in a way that ultimately regulates overall fitness (Vallejo-Marín *et al*., 2010; Eckert *et al*., 2016). The ability to reproduce sexually and asexually (through modular growth or asexual propagules) has ecological and evolutionary advantages. Modular growth, the repeated addition of vegetative structures or subunits (e.g. shoots, polyps), allows individuals to rapidly expand within the local habitat and capture resources, eliminates the need for a mate, reduces the likelihood of a genotype dying and avoids the costs of sex (Vallejo-Marín *et al*., 2010; Barrett, 2015). Localized asexual reproduction and recruitment can, however, come at a cost as increased levels of clonality reduce the opportunity for mates and outcrossed sexual reproduction, which can lead to an overall decline in fitness if a substantial part of the reproduction relies on sexuality (Van Drunen *et al*., 2015). The relationship is complex and can be influenced by ecological and environmental factors, varying within and between species (Silvertown, 2008). Isolation from potential mates, competition, unfavourable conditions for seedling recruitment, disturbance levels and immigration rates will ultimately determine levels of genotypic diversity (Vallejo-Marín *et al*., 2010; McMahon *et al*., 2014; Oliva *et al*., 2014; Connolly *et al*., 2018).

Patterns of recruitment can determine the structure of clonal populations (Eriksson, 1993). Many clonal organisms employ a repeated seedling recruitment strategy where continued sexual reproduction or immigration leads to high genotypic diversity within a population (Eriksson, 1993). Repeated seedling recruitment is prominent in heterogeneous and changing environments, creating opportunities for recruits to establish and leading to high genotypic diversity within a population. (Silvertown, 2008; McMahon *et al*., 2014; Manent *et al*., 2020). This in turn promotes sexual reproduction where the production of novel genotypes favours adaptation to spatial and/or temporal variation in conditions (Williams, 1975; Eriksson, 1989). Even if recruitment is rare, repeated seedling recruitment should lead to populations with high levels of genotypic diversity as new recruits continue to establish through local production and immigration as established genotypes are lost to disturbance.

In contrast to repeated seedling recruitment, initial seedling recruitment reflects survivorship of the initial cohort of recruits over time (Eriksson, 1993). Populations dominated by a small number of genotypes are often characterized by an initial seedling recruitment strategy during population establishment, followed by intraspecific competition between genotypes as recruits grow until equilibrium is reached between clonal density, resource availability and local adaption (Eriksson, 1993). In this case, genotypic diversity will be eroded over time as the fittest genotypes dominate. In stable, homogeneous environments, initial seedling recruitment strategies are common where the fittest genotypes monopolize resources and outcompete less fit genotypes or where dispersal costs are high clonal expansion through modular growth, or localized recruitment of asexual propagules will be favoured (Sherman *et al*., 2007; Silvertown, 2008; Eckert *et al*., 2016). In extreme cases, a few successful genotypes may come to dominate, reducing the number of mates available for outcrossing and leading to the complete loss of sexual reproduction (Dorken and Eckert, 2001; Honnay and Jacquemyn, 2008; Sherman and Ayre, 2008; Smith *et al*., 2018). An initial seedling recruitment strategy is also common in isolated populations and those established by a single or small number of recruits that rely on asexual reproduction or modular growth to persist in the absence of new recruits or mates (Smith *et al*., 2018). Variations in recruitment strategy are determined by disturbance regimes, environmental conditions, isolation and dispersal potential (Becheler *et al*., 2010; McMahon *et al*., 2014; Eckert *et al*., 2016; Connolly *et al*., 2018).

Dispersal and recruitment are important processes that can significantly influence patterns of genetic differentiation and levels of genetic and genotypic diversity. In clonal (modular) organisms, gene flow can potentially occur through the dispersal and recruitment of either sexual or asexually produced propagules, including modular fragments (Erftemeijer *et al*., 2008; Källström *et al*., 2008). The dispersal of propagules beyond local populations can increase genotypic richness by introducing new alleles and genotypes into populations. Dispersal potential increases with increasing genet production but recruitment decreases as modular density increases (Oliva *et al*., 2014). In marine environments hydrodynamic processes have been shown to play a major role in gamete and propagule dispersal (both sexual and asexual), which can affect levels of genotypic diversity and population structure (Kamel *et al*., 2012; Sinclair *et al*., 2014*a*, *b*, 2016).

Seagrasses are habitat-forming marine angiosperms that are ecologically and economically valuable, but are declining globally (Orth *et al*., 2006; Dunic *et al*., 2021). Seagrasses display wide variation in the relative importance of sexual and clonal reproduction, within and between species, and over local and broad scales ranging from exclusively sexual to entirely clonal (Arnaud-Haond *et al*., 2020). Genotypic and genetic diversity in seagrass populations can affect population resilience but may depend on disturbance regimes or environmental conditions (McMahon *et al*., 2014; Connolly *et al*., 2018). High genotypic and genetic diversity can lead to greater adaptability under stress (Hughes and Stachowicz, 2004) whereas under stable or stressful conditions a few well-adapted genotypes may come to dominate populations (Arnaud-Haond *et al*., 2010; Oliva *et al*., 2014; Connolly *et al*., 2018). Recent research has shown the size and intensity of physical disturbance may dictate the relationship between resilience and genotypic diversity (McMahon *et al*., 2014; Macreadie *et al.*, 2014*a*). In a seascape context, the balance between sexual reproduction, clonal reproduction and modular growth will be important to maintain populations into the future.

The seagrass *Heterozostera nigricaulis* (syn. *Heterozostera tasmanica*, *Zostera nigricaulis*) is common in bays and inlets throughout the temperate waters of southern Australia where it is an important habitat for fauna (Jenkins and Wheatley, 1998; Smith *et al*., 2008), and contributes to carbon sequestration (Macreadie *et al*., 2014*b*) and sediment stabilization (Bulthuis *et al*., 1984). *Heterozostera nigricaulis* produces small negatively-buoyant seeds with limited dispersal potential, but seeds may be dispersed over large distances by detached fragments (Kendrick *et al*., 2012; Sherman *et al*., 2018; Smith *et al*., 2022). Like all seagrasses *H. nigricaulis* can use rhizome extension to undergo modular expansion within a habitat, but also produces aerial asexual propagules that grow horizontally off the main stem of the parent plant and readily detach and disperse in the water (Cambridge *et al*., 1983; Thomson *et al*., 2015). In this study we use direct estimates of reproductive investment (flowering and seed production), population genetic surveys and a biophysical model to assess the relationship between connectivity and the relative importance of sexual and asexual reproduction in maintaining populations. We predict that populations with high levels of connectivity and investment in flowering and seed production will display higher levels of genotypic diversity, while more isolated populations with lower investment in flowering and seed production will display higher levels of clonality.

## MATERIAL AND METHODS

### Sample collections

**Fig. 1.**
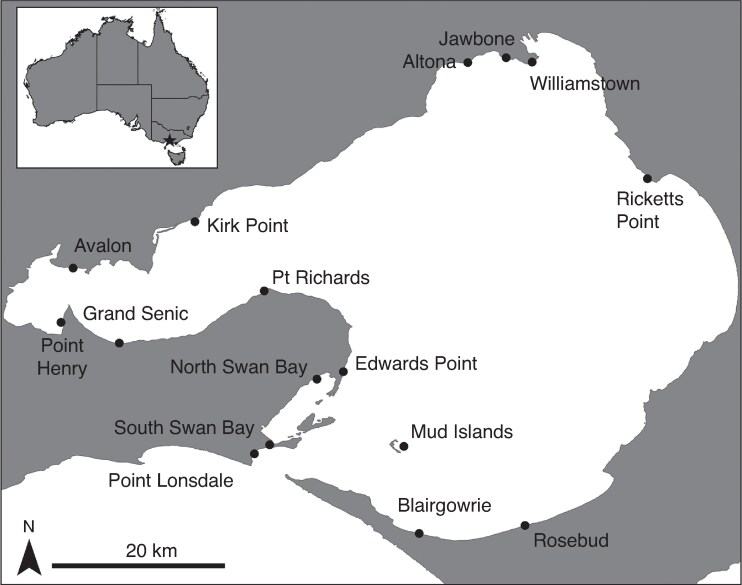
Genetic and reproductive *Heterozostera nigricaulis* sampling sites in Port Phillip Bay.

Seagrass samples were collected from 16 sites in Port Phillip Bay, south-eastern Australia (spring 2011), to assess levels of genetic diversity, clonality and connectivity (Fig. 1; Supplementary Data Table S1). Port Phillip Bay is a large (1933 km^2^) semi-enclosed bay in south-eastern Australia where the seagrass *H. nigricaulis* is the dominant structured subtidal habitat. Sites were distributed across seagrass meadows around the bay representing a range of habitat conditions and spatial spread. At each site 45 samples were collected for genetic analysis from across three 10 × 10-m quadrats (15 per quadrat). Samples within quadrats were haphazardly collected, leaving at least 1 m between samples to minimize the collection of shoots from the same ramet. Quadrats were 50–200 m apart. Each sample consisted of 10 cm of leaf obtained from a single stem. Samples were placed in individual bags on ice before being frozen in the laboratory at −20 °C in a freezer.

### DNA extraction and genotyping

Genomic DNA was isolated from leaf tissue using DNeasy plant kits (Qiagen) following the manufacturer’s instructions. All samples were genotyped using nine polymorphic microsatellite markers previously developed for this species: ZosVic49, ZosVic50, ZosVic55, ZosVic60, ZosVic66, ZosVic68, ZosVic69, ZosVic70b and ZosVic71 (Smith *et al*., 2013). Microsatellites were amplified using a polymerase chain reaction (PCR) conducted in 11-µL volumes containing: 10 ng of genomic DNA, 5 µL PCR Master Mix (Qiagen, USA) and 4 µL primer multiplex (0.26 µm of each forward primer and fluorescent dye, 0.13 µm of reverse primer). Thermal cycling conditions for the PCR were: initial hot start at 94 °C for 15 min; ten cycles of 94 °C for 45 s, 55 °C for 45 s and 72 °C for 45 s; ten cycles of 94 °C for 45 s, 53 °C for 45 s and 72 °C for 45 s; 20 cycles of 94 °C for 45 s, 50 °C for 45 s and 72 °C for 45 s; and final elongation at 72 °C for 15 min. PCR amplicons were electrophoresed using an ABI 3130xl Genetic Analyzer, incorporating LIZ 500 (−250) size standard (Applied Biosystems). Alleles were scored using GENEMAPPER, v.3.7 (Applied Biosystems).

### Genetic analyses

To assess the independence of loci for genetic analysis, pair-wise combinations of loci were tested for linkage disequilibrium within each quadrat at each site to determine the independence of loci using GENEPOP v.4.2 (Rousset, 2008). Only unique multilocus genotypes were used for linkage disequilibrium as clonal reproduction can create non-random associations between loci mimicking physical linkage. No consistent evidence of linkage between any loci across different sites was detected. From a total of 1446 possible pairwise tests only six were significant after the application of a sequential Benjamini–Hochberg correction and these were not consistent across sites. Potential null alleles at each locus within each site were assessed using MICROCHECKER (Van Oosterhout *et al*., 2004). Both ZosVic71 (14 sites recording null alleles) and ZosVic66 (13 sites having null alleles) consistently showed the presence of null alleles across the 16 survey sites. Null alleles were found at five sites for ZosVic60. ZosVic68 and ZosVic55 showed the presence of null alleles at three sites and ZosVic49 had null alleles at two sites. There was no evidence of null alleles for ZosVic69 and ZosVic70b. To assess if populations conformed to Hardy–Weinberg equilibrium, probabilities were calculated using Markov chain parameters of 1000 iterations per batch using a Hardy–Weinberg Exact Test in GENEPOP v.4.2 (Rousset, 2008). After application of sequential Benjamini–Hochberg correction for multiple tests only 50 from a total of 379 tests were out of Hardy–Weinberg equilibrium. Of the 50 tests that were out of Hardy–Weinberg equilibrium, 20 were from the locus ZosVic71 and 19 from the locus ZosVic66. These results support the presence of null alleles at these loci at some sites. Despite the possible presence of null alleles, these loci were included in analyses to allow the identification of more unique multilocus genotypes when assessing genotypic diversity and excluding it did not change the overall estimates of population structure (Supplementary Data Table S3).

### Estimates of genotypic diversity and the detection of multilocus genotypes

Levels of genotypic diversity across and within sites was determined by comparing the number of unique multilocus genotypes (*G*) detected to the number of samples collected within each site (*N*). Identical multilocus genotypes can occur through resampling of the same clone (modular growth), the recombination of the same alleles in different individuals, somatic mutation or scoring error. The probability of identical multilocus genotypes arising from different sexual reproductive events was assessed using *P*_sex_ using the statistical software GENCLONE (Arnaud-Haond and Belkhir, 2007). *P*_sex_ values ranged between <0.001 and 0.121, indicating that sampling the same multilocus genotype from separate sexual events was low. The *P*_sex_ value of 0.121 was recorded at Point Lonsdale where only nine unique multilocus genotypes from a total of 45 samples were recorded, increasing the *P*_sex_ value. Excluding Point Lonsdale, the highest *P*_sex_ value was 0.0001. Therefore, individuals with an identical multilocus genotype were considered clones. Genotypic diversity [*R* = (*G* − 1)/*N* − 1, where *G* = number of unique multilocus genotypes, was calculated in Genodive (Meirmans and Van Tienderen, 2004)]. The presence of identical multilocus genotypes across multiple sites was assessed using *P*_sex_ in GENCLONE (Arnaud-Haond *et al*., 2007). Identical multilocus genotypes at difference sites may result from the dispersal and recruitment of asexual propagules or separate sexual events (Cambridge *et al*., 1983; Thomson *et al*., 2015).

### Patterns of genetic diversity and population structure

Patterns of genetic diversity across sites were expressed as the mean number of alleles per site (*Na*), and observed (*H*_O_) and expected heterozygosity (Nei’s unbiased estimate *H*_E_) using the program ARLEQUIN (Excoffier and Lischer, 2010). Analyses were calculated using only unique multilocus genotypes as clonal reproduction may influence estimates of population structure. Estimates of population structure and patterns of connectivity were estimated as standardized F-statistics and pairwise *F*_ST_ between sites calculated in FSTAT 2.9.3.2 (Goudet, 2002) and significance levels adjusted to 0.0004 to account for multiple comparisons across sites using the Bonferroni correction (Rice, 1989). Isolation by distance calculations were used to assess the relationship between both geographic and genetic connectivity. Pairwise *F*_ST_/(1 − *F*_ST_) was compared to across-water geographical distances (the shortest route across water between sites in km) using a MANTEL test (Sokal, 1979) with 9999 permutations in the program GenALEx 6.5 (Peakall and Smouse, 2006). All variables were log transformed to meet assumptions of normality. Principal components analysis was done using the package ‘adegenet’ in R to visualize genetic structuring across populations.

### Flowering and seed production

As a measure of investment in sexual reproduction, we assessed seed and spathe abundance at each of the 16 sites sampled for genetic analysis. Pre-published data from two previous studies of these sites was used to determine flowering and seed investment at each site (Smith *et al*., 2016*a*, *b*) with the addition of Point Lonsdale sampled within this study sampling period. Methodology followed published protocols (Smith *et al*., 2016*a*, *b*).

### Hydrodynamic modelling

An existing biophysical dispersal model (Treml *et al*., 2015; Fobert *et al*., 2019) was parameterized to quantify the dispersal potential among all seagrass sites within Port Phillip Bay and to assess the influence on gene flow and genotypic diversity. The biophysical dispersal model uses spatial data describing the geographical structure of the bay and the location and quality of seagrass habitat (Blake and Ball, 2001), augmented by field observations and validation in this study. This 3D hydrodynamic model of the bay has been well validated through a series of projects since the late 1980s (Black *et al*., 1991, 1993; Lee *et al*., 2012; Fobert *et al*., 2019), resulting in an accurate and high-resolution (400 m horizontal resolution, eight vertical layers, hourly data) depiction of currents in the bay.

To develop quantitative predictions of seagrass dispersal, we released 1000 virtual seed-carrying fragments from all available habitat patches (Blake and Ball, 2001) every 3 h between 1 October and 31 December (totalling more than 700 000 virtual fragments per patch per year), the period when seeds are mature on plants (Smith *et al*., 2016*b*), for all years of data collection (2009–2011). The relative reproductive output originating from each patch was determined by the size of each habitat patch. All seagrass fragments were allowed to drift for a maximum of 28 d, the period of time fragments carrying seeds remained buoyant for the closely related *Zostera marina* (Harwell and Orth, 2002). Fragments were positive for the first week, neutral for the second week and negatively buoyant for the last 2 weeks with fall velocities of 0.0049, 0.00 and −0.005 respectively (Harwell and Orth, 2002). Settlement was allowed to occur after the initial 6 h when virtual fragments came in contact with the seabed. Mortality of fragments increased through time, from 25 % loss per week in the first week to 100 % mortality at 4 weeks (Harwell and Orth, 2002). Mortality also occurred when fragments reached the shoreline – these beached virtual fragments were effectively removed from the simulation. The biophysical model produces a probability matrix representing the likelihood that virtual fragments released in source patches (rows) arrive and settle in destination patches (columns). This dispersal probability was converted to a flow matrix (Treml *et al*., 2012) representing the relative number of fragments arriving into a destination patch (column) that came from each source patch (row). This matrix was produced by multiplying the probability matrix by the patch area for each source site to give a more accurate representation of the flow of propagules between sites. Connectivity data for our 16 sample sites were extracted from this flow matrix and used in all subsequent analyses.

### Network analysis

A graph-based theory network was used to visualize the genetic connections between sites, where sampling sites are represented as nodes and the genetic similarity (*F*_ST_) is represented as edges between nodes in the network (Kivelä *et al*., 2015). Three local-scale network properties were quantified for this analysis: (1) the node-degree, quantifying the number of other nodes linked with each node, (2) betweenness centrality, measuring the number of shortest paths which pass through each node, and (3) relative inflow metric, which sums the total relative incoming propagules to a destination patch. The network was built using EDENETWORKS software (Kivelä *et al*., 2015) using *H. nigricaulis* genotypes from each site as nodes connected by links based on pairwise *F*_ST_ estimates. The threshold for inclusion in the network was set to 0.04, the lowest value that connected all nodes in a network, with the exception of Point Lonsdale, which was genetically isolated. All connections above this threshold were removed from the network.

### Statistical analysis

A Pearson’s correlation analysis was used to explore the relationship between sexual reproductive investment (genotypic diversity, spathe density, seed density), genetic diversity (number of alleles and expected heterozygosity) and population connectivity (node-degree, betweenness centrality and total inflow). To understand the relative importance of the different factors driving levels of genotypic diversity at a site, a beta regression analysis (Douma and Weedon, 2019) was run in the ‘betareg’ package (Cribari-Neto and Zeileis, 2010) in R Studio (R Core Team, 2020). Final model selection was achieved using beta regression with a logit link using the dredge function in the ‘MuMIm’ package (Barton, 2020). Point Lonsdale was removed from the final analysis due to the absence of flowering and spathe data. Spathe density, node-degrees and expected heterozygosity were not included in the analysis because of collinearity with another variable (Supplementary Data Table S3). Seed density (seeds m^−2^) and total relative inflow of propagules each patch receives from all other patches calculated from the flow matrix were log transformed because of the large range of values. A Mantel test was used to directly compare genetic connectivity (*F*_ST_ values) and dispersal distance. In this case, dispersal strength was determined as the number of propagules given and received between two sites from the flow matrix. Mantel tests were completed using the IBD function in GenALEx 6.5 using *F*_ST_/1 − *F*_ST_ and all variables were log transformed to meet assumptions of normality.

## RESULTS

### Reproductive investment

#### Flowering and seed production

**Fig. 2.**
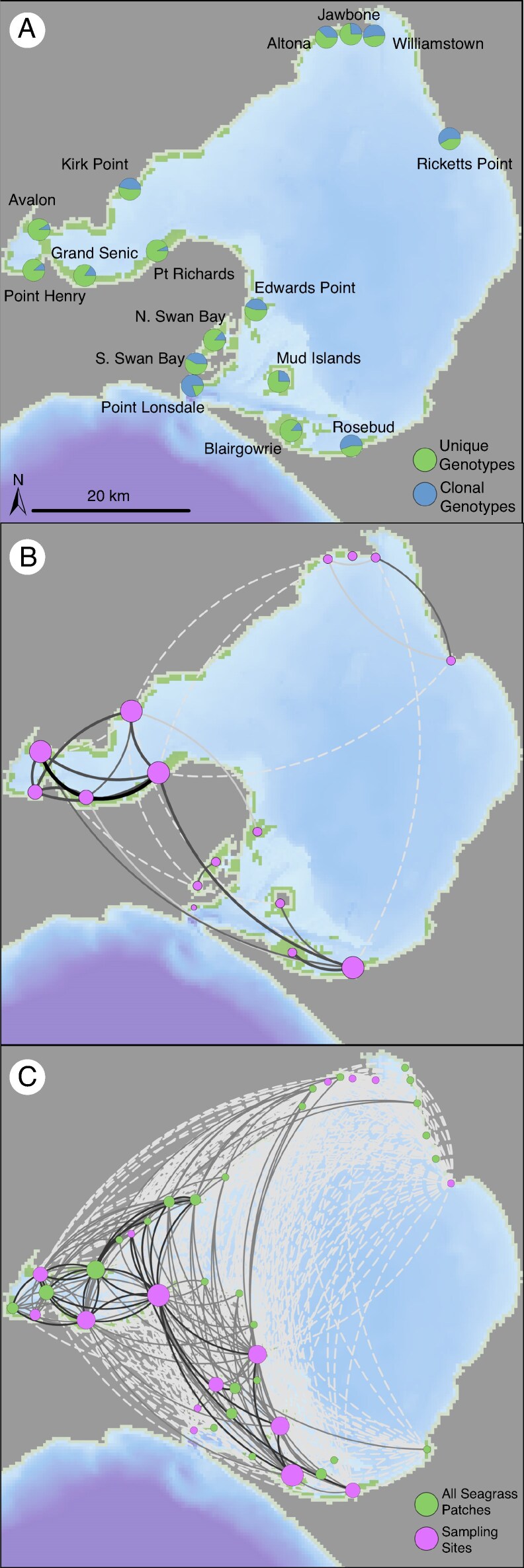
Proportion of unique and clonal *Heterozostera nigricaulis* genotypes at each sampling site in Port Phillip Bay (A). Connectivity of *H. nigricaulis* populations in Port Phillip Bay using genetic network analysis based of *F*_ST_ values (B) and biophysical modelling (C). The size of each node represents the number of connections (node degree) and the weight of the connection is relative to the flow between patches from the biophysical model. The direction of flow in the biophysical network (bottom, C) can be read by following the arc in a clockwise direction. The threshold for inclusion in the network was set at 0.04. Seagrass patches are represented by green areas in the maps (Blake and Ball, 2001).

Flowering and seed production varied widely among the 16 sites (Table 1). The mean (±s.e.) spathe density across all sites was 1041.38 ± 254 m^−2^, but this varied from only 7.38 spathes m^−2^ at Jawbone to 270.80 spathes m^−2^ at North Swan Bay. Seed bank density also showed substantial variation among sites and was strongly correlated with spathe density (Pearson correlation *r* = 0.614, *P* = 0.015). Across all sites mean (±s.e.) seed bank density was 2185.32 ± 723 seeds m^−2^, with Edwards Point having only 14.15 seeds m^−2^ compared to 9140.83 seeds m^−2^ at Point Henry (Table 1; Fig. 2). Northern Port Phillip Bay (Ricketts Point, Williamstown, Jawbone, Altona) produced lower seed and spathe densities than all other regions (four of the five lowest densities) while sites in the Western Geelong Arm (Avalon, Point Henry, Grand Scenic) produced over 50 % of the seed and 40 % of the spathe density in Port Phillip Bay.

**Table 1.** Summary of values for each site.

	Reproductive		Clonality			Genetic			Connectivity		
	Seed density (seeds m^−2^)	Spathe density (spathes m^−2^)	*N*	UMLGs	*R*	*Na*	*H*_E_	*H*_O_	Degree	BC	Relative inflow
Ricketts Point	56.61	14.76	45	19	0.41	5.22	0.60	0.60	3	2.16	1.01
Williamstown	350.32	105.13	45	21	0.45	5.11	0.61	0.56	3	2.67	1.18
Jawbone	28.31	7.38	42	31	0.73	5.56	0.66	0.57	1	0	1.48
Altona	24.06	10.45	45	28	0.61	6.22	0.62	0.62	3	4.17	0.97
Kirk Point	629.22	629.42	45	24	0.52	5.56	0.68	0.59	8	41.50	9.47
Avalon	4856.18	2611.73	45	41	0.91	6.33	0.63	0.56	6	5.83	9.92
Point Henry	9140.83	1316.32	45	40	0.89	5.89	0.63	0.49	4	0	1.58
Grand Scenic	3269.63	2311.09	45	38	0.84	5.67	0.61	0.64	5	0.67	11.79
Point Richards	987.97	1176.76	43	40	0.93	6.22	0.63	0.56	7	20.50	17.68
Edwards Point	14.15	247.16	45	25	0.55	4.89	0.60	0.63	1	0	4.54
North Swan Bay	3227.16	2705.80	45	39	0.86	6.33	0.60	0.56	1	0	4.55
South Swan Bay	1241.32	1167.53	43	25	0.57	5.67	0.63	0.49	2	13.00	0.79
Point Lonsdale	–	–	45	9	0.18	2.78	0.42	0.64	0	0	0.15
Mud Islands	806.79	31.36	44	33	0.74	5.89	0.65	0.56	2	0	4.14
Blairgowrie	6933.09	1706.11	45	39	0.86	5.56	0.64	0.58	1	0	0.44
Rosebud	1214.17	1579.77	45	20	0.43	6.44	0.64	0.55	7	24.50	0.38
Mean	2185.32	1041.38	712	472	0.66	5.58	0.62	0.57			

Seed and spathe data were not collected at Point Lonsdale.

*N* = number of samples, UMLGs = number of unique multilocus genotypes, *R* = genotypic diversity, *Na* = number of alleles, *H*_E_ = expected heterozygosity, *H*_O_ = observed heterozygosity, Degree = number of connections in network analysis, BC = betweeness centrality, Inflow = the relative number of propagules received in the biophysical model.

#### Estimates of clonal structure

A total of 712 samples were genotyped across the 16 sites, with 472 unique multilocus genotypes detected across the entire data set. Mean genotypic diversity was moderate (*R* = 0.66 ± 0.06 s.e.) but varied widely between sites (Table 1). Point Lonsdale had the lowest genotypic diversity (*R* = 0.18) and Point Richards had the highest (*R* = 0.93). At Avalon, Point Richards and Point Henry most genets were represented by a single ramet, and across these sites the largest genets only occurred three times each. In contrast, Point Lonsdale had only nine genotypes present, with the largest genet accounting for 55 % of ramets across all three quadrats at that site, and Williamstown, Rickets Point and Rosebud all had clones represented by ≥15 % of ramets and relatively few genets with only a single ramet. Three identical multilocus genotypes were shared between Mud Island and Rosebud (*P*_sex_ = 3.45 × 10^−5^, 3.57 × 10^−5^ and 1.51 × 10^−8^) and a single genotype was shared between Jawbone and Williamstown (*P*_sex_ = 2.21 × 10^−6^).

### Patterns of genetic diversity and population structure

We detected high levels of genetic diversity across sites in Port Phillip Bay. The mean number of alleles across all loci and all sites was 5.58 ± 0.94 s.e. (Table 1). The number of alleles ranged from three to 16 across individual loci and observed and expected heterozygosity ranged from 0.063 to 0.893 and from 0.153 to 0.857 respectively (Supplementary Data Table S2). Point Lonsdale had the lowest mean number of alleles, 2.78 ± 0.49 s.e., and Rosebud had the highest, 6.44 ± 0.94 s.e. Overall mean expected heterozygosity was 0.62 ± 0.08 s.e. and ranged from 0.42 ± 0.09 s.e. at Point Lonsdale to 0.68 ± 0.07 s.e. at Kirk Point. Observed heterozygosity was lower than expected heterozygosity with a mean of 0.57 ± 0.08 s.e. across all sites. The lowest observed heterozygosity was at South Swan Bay, 0.49 ± 0.09 s.e., and the highest at Grand Scenic, 0.64 ± 0.09 s.e. (Table 1).

There was significant genetic structuring among sites sampled in Port Phillip Bay. Global *F*_ST_ was 0.061 ± 0.01 s.e. (95 % CI = 0.06–0.07), but pairwise comparisons between sites indicated significant variation in levels of gene flow and connectivity among sites. Of a total of 120 pairwise comparisons, only 17 were not significantly differentiated from each other (Table 2), even using a very conservative significance threshold (*P* < 0.0004). Eight of the non-differentiated comparisons occurred between sites in the Geelong arm, and a further six were associated with Rosebud. Point Lonsdale, Edwards Point and North Swan Bay were the most structured sites, with all being significantly differentiated from all other sites. In particular, Point Lonsdale showed the highest levels of genetic differentiation from other sites, indicating that this site is largely isolated from all others.

**Table 2.** Pairwise *F*_ST_ (bottom matrix) and relative inflow values comparing sites within Port Phillip Bay (top matrix).

	Ricketts Point	Williamstown	Jawbone	Altona	Kirk Point	Avalon	Point Henry	Grand Scenic	Point Richards	Edwards Point	North Swan Bay	South Swan Bay	Point Lonsdale	Mud Islands	Blairgowrie	Rosebud
Ricketts Point		0.027	0.028	0.030	0.020	0.007	0.001	0.003	0.018	0.084	0.003	0	0.002	0.020	0.019	0.015
Williamstown	0.021		0.041	0.031	0.025	0.009	0.001	0.012	0.043	0.101	0.003	0	0.002	0.059	0.079	0.029
Jawbone	***0***.***055***	***0***.***050***		0.049	0.023	0.010	0.001	0.020	0.053	0.153	0.003	0	0.003	0.087	0.110	0.030
Altona	0.030	***0***.***028***	***0***.***079***		0.020	0.011	0	0.018	0.029	0.107	0.003	0	0.002	0.065	0.070	0.020
Kirk Point	***0***.***036***	***0***.***039***	***0***.***044***	***0***.***032***		0.175	0.087	0.737	1.885	0.265	0.026	0	0.007	0.148	0.172	4.975
Avalon	***0***.***065***	***0***.***068***	***0***.***056***	***0***.***087***	0.034		1.160	4.037	1.221	0.018	0.002	0	0	0.006	0.006	0.003
Point Henry	***0***.***052***	***0***.***066***	***0***.***067***	***0***.***055***	0.012	0.017		0.657	0.229	0	0	0	0	0.001	0.001	0
Grand Scenic	***0***.***067***	***0***.***062***	***0***.***055***	***0***.***080***	0.024	0.003	0.015		10.297	0.908	0.073	0	0.017	0.261	0.249	0.127
Point Richards	***0***.***033***	***0***.***049***	***0***.***040***	***0***.***070***	0.015	***0***.***016***	***0***.***018***	0.010		3.104	0.258	0	0.060	1.182	1.278	0.762
Edwards Point	***0***.***056***	***0***.***061***	***0***.***071***	***0***.***063***	***0***.***029***	***0***.***072***	***0***.***073***	***0***.***056***	***0***.***052***		0.598	0	0.061	0.591	1.649	0.756
North Swan Bay	***0***.***068***	***0***.***092***	***0***.***093***	***0***.***082***	***0***.***061***	***0***.***073***	***0***.***065***	***0***.***063***	***0***.***060***	***0***.***080***		1.752	0.014	0.050	0.073	0.011
South Swan Bay	***0***.***071***	***0***.***078***	***0***.***092***	***0***.***080***	0.037	***0***.***053***	***0***.***055***	***0***.***052***	***0***.***044***	***0***.***078***	***0***.***023***		0	0	0	0
Point Lonsdale	***0***.***219***	***0***.***182***	***0***.***190***	***0***.***181***	***0***.***161***	***0***.***174***	***0***.***196***	***0***.***165***	***0***.***163***	***0***.***122***	***0***.***154***	***0.125***		0.040	0.124	0.019
Mud Islands	***0***.***085***	***0***.***083***	***0***.***050***	***0***.***099***	***0***.***047***	***0***.***036***	***0***.***056***	***0***.***042***	***0***.***033***	***0***.***053***	***0***.***073***	***0.054***	***0***.***138***		2.392	0.798
Blairgowrie	***0***.***080***	***0***.***082***	***0***.***080***	***0***.***093***	***0***.***071***	***0***.***062***	***0***.***093***	***0***.***073***	***0***.***062***	***0***.***092***	***0***.***090***	***0.079***	***0***.***180***	***0***.***066***		0.349
Rosebud	0.036	0.031	***0***.***039***	***0***.***051***	0.031	***0***.***030***	***0***.***047***	***0***.***034***	0.019	***0***.***046***	***0***.***056***	***0.043***	***0***.***150***	0.027	0.018	

Significant values for *F*_ST_ are shown in bold and italics when *P* < 0.0004 to account for multiple tests. Colours indicate the level of connectivity: darker colours have high connectivity and lighter colours have low connectivity.

Network analysis of genetic data using *F*_ST_ values revealed strong connections within the Geelong Arm. Kirk Point had the most node degrees of any site (eight) and five of the six most connected sites were in the Geelong Arm (Table 1; Fig. 2). Kirk Point had the highest betweenness centrality (41.5) followed by Rosebud (24.5) and Point Richards (20.5), suggesting these sites belong to important stepping-stone areas for dispersal and gene flow. Point Lonsdale was isolated from all other sites, not receiving immigrants or acting as a source to other sites. Edwards Point, North Swan Bay, Jawbone and Blairgowrie were also largely isolated from the other sites, recording one and two degrees respectively.

**Fig. 3.**
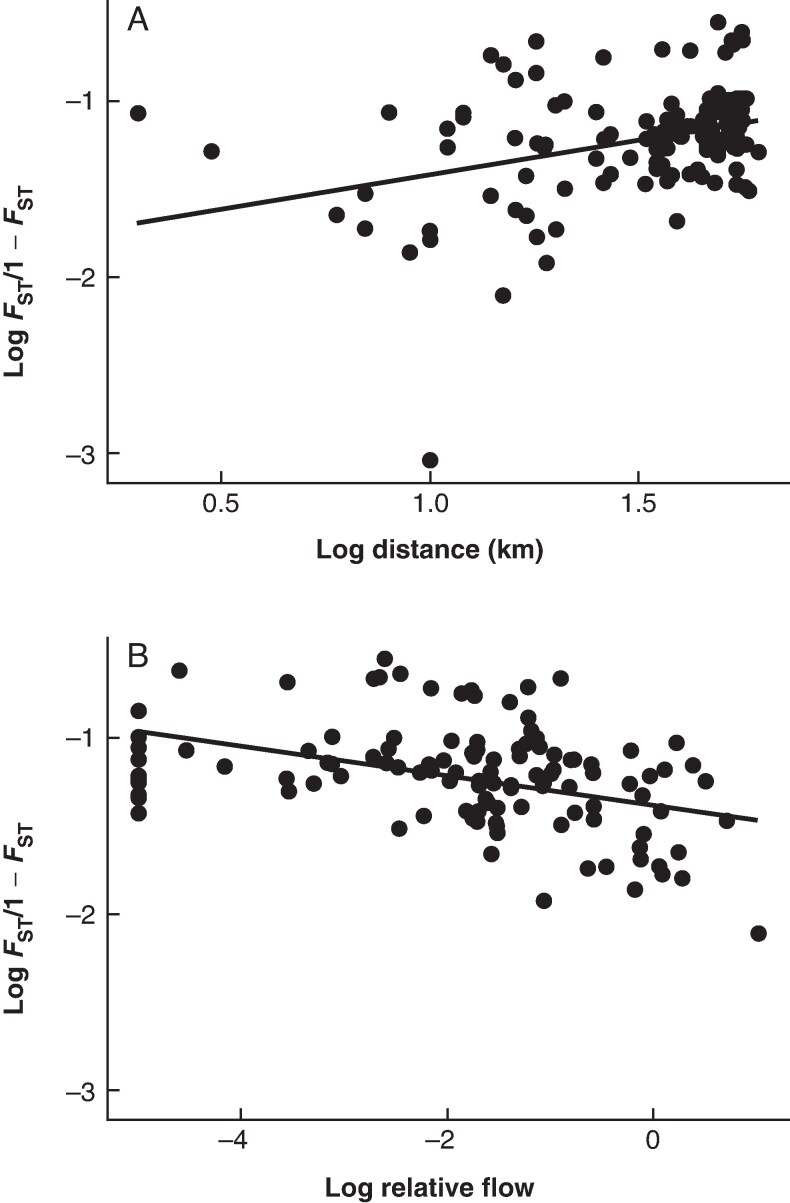
Relationship between *F*_ST_/1 − *F*_ST_ and geographical distance (over water) (B) and propagule inflow (B) between sites for *Heterozostera nigricaulis* in Port Phillip Bay (*R*^2^ = 0.12). All data were log-transformed to meet assumptions of normality.

Our test for isolation by distance was significant (*P* < 0.001), but the analysis only explained a relatively small proportion of the total variance (*R*^2^ = 0.12), indicating that logged Euclidian distance between sites is not a good predictor of connectivity (Fig. 3). Mantel tests also found a significant relationship (*P* = 0.039) between logged genetic differentiation and the logged dispersal probability based on hydrodynamic modelling. The analysis again only accounted for a small amount of the total variance but was greater than Euclidian distance (*R*^2^ = 0.15). Principal components analysis showed little differentiation between sites with the exception of Point Lonsdale that was separated from the other sites (Supplementary Data Fig. S1).

### Hydrodynamic connectivity

All seagrass patches in Port Phillip Bay received propagules in the biophysical flow matrix, but there was two orders of magnitude variation in the number of propagules received across destinations. Of the 16 sites used in the analysis, Rosebud received the fewest propagules in the relative flow matrix (0.38 inflow) compared to Point Richards, which received the greatest number of propagules (17.68 inflow; Table 1; Fig. 2). Patches in the west and south of the bay had greater connectivity than those in the north of Port Phillip Bay. Patches in the western Geelong Arm had high connectivity within this part of the bay but had lower propagule exchange with patches in the rest of the bay.

### Comparisons of reproductive investment, genetic diversity and connectivity

**Fig. 4.**
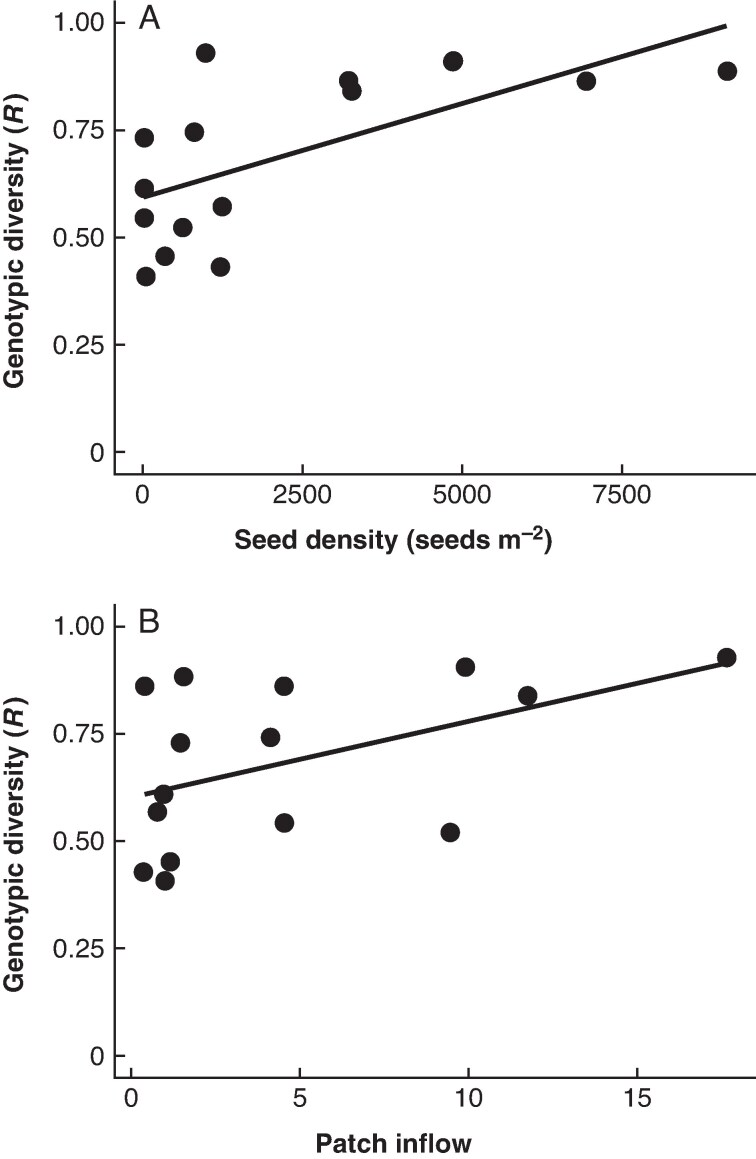
Relationship between genotypic diversity and seed bank density (A) and relative patch inflow (B) across sites in Port Phillip Bay. Pseudo-*R*^2^ = 0.73.

Correlation analysis showed significant relationships between several estimates of reproductive investment, genetic diversity and connectedness among sites (Supplementary Data Table S3). Sites with high genotypic diversity were strongly correlated with flowering and seed production, levels of genetic diversity and levels of connectivity (Table S3). Beta regression analysis showed a significant positive relationship between levels of genotypic diversity (*R*) and both seed density and the total inflow of propagules at each site (pseudo *R*^2^ = 0.73; Table 3; Fig. 4).

**Table 3.** Results from beta regression analysis comparing the relationship between genotypic diversity (*R*) and seed density and total relative inflow of propagules each site receives for the seagrass *Heterozostera nigricaulis* from Port Phillip Bay, Australia.

Source	Estimate	s.d.	*Z* value	*P*
Intercept	−0.035	0.187	−0.186	0.852
**Seed bank density**	<0.001	<0.001	4.083	**<0**.**001**
**Total inflow**	0.093	0.029	3.253	**0**.**001**

Significant values are shown in bold type.

## DISCUSSION

Our reproductive and genetic surveys of the seagrass *H. nigricaulis* showed that the relative investment in sexual and asexual reproduction varied widely between sites within Port Phillip Bay. Estimates of hydrodynamic connectivity between sites showed that less-connected sites showed higher clonality (i.e. lower genotypic diversity). In contrast, sites that were more connected showed higher levels of genotypic diversity and greater investment in flowering and seed production (a direct estimate of sexual reproduction). Seed banks are replenished annually (Smith *et al*., 2016*b*) and provide the basis for high genotypic diversity by providing the opportunity for continued recruitment, in line with repeated seedling recruitment strategies. Small-scale disturbances (algal blooms, seasonal die off, herbivory, etc.) facilitate space and resources for new recruits to maintain persistent seagrass populations (McMahon *et al*., 2014; Oliva *et al*., 2014).

Our results suggest that dispersal and connectivity between populations is a major contributor to levels of genotypic diversity. Sites that had the greatest inflow of propagules as predicted from the biophysical model had higher levels of genotypic diversity than sites that received few propagules. Hydrodynamic features play an important role in determining connectivity in seagrass systems at both local (Zipperle *et al*., 2011; Kamel *et al*., 2012; Sinclair *et al*., 2014*b*) and broad seascape scales (Ruiz-Montoya *et al*., 2012; Nakajima *et al*., 2014; Ruiz-Montoya *et al*., 2015). Sites with high hydrodynamic connectivity will receive genotypes from other populations, increasing the number of unique recruits and subsequently levels of genotypic diversity (Thomson *et al*., 2015). Genetic structuring (*F*_ST_) was low between sites with high dispersal probabilities, suggesting there is dispersal and establishment of recruits between these sites (Fig. 2). Highly connected sites with high seed production and genotypic diversity not only are likely to persist locally, but they are also exporting seeds and genes to other populations.

Highly clonal sites, on the other hand, had low seed densities and were less connected, based on genetic and hydrodynamic connectivity, with other sites. At isolated sites, successful colonization may be hindered by distance and habitat suitability, ensuring recruitment of immigrants is low and initial recruits are able to monopolize resources, resulting in low genotypic diversity (Nathan, 2006; Silvertown, 2008). Initial colonizers or well-adapted clones can outcompete newer or smaller recruits, thereby diminishing the probability of successful sexual reproduction and recruitment (Silvertown, 2008; Eckert *et al*., 2016). While few plant populations are so isolated that they have entirely lost the capacity for sexual reproduction, at smaller scales like those observed in Port Phillip Bay, genets within isolated populations tend to allocate resources toward modular growth and expansion to persist under unfavourable conditions, shifting their allocation toward sexual reproduction during more favourable periods. In one of the few studies that directly compared reproduction strategies of modular plants under varying environmental conditions, Wang *et al*. (2018) found the cost of sexual reproduction was much greater in highly stressed environments compared with less stressed conditions. In Port Phillip Bay, the more clonal populations are located in isolated habitats where environmental conditions restrict suitable space for seagrass growth and resources are likely to be limited (Hirst *et al*., 2017; Hirst and Jenkins, 2017). Under these conditions large well-established clones are able to dominate by allocating less energy to sexual reproduction and more energy to modular growth and clonal expansion (Diaz-Almela *et al*., 2007; Connolly *et al*., 2018). At these sites where clones are larger, successful outcrossing decreases and competition for space is higher, increasing the benefits of modular growth as accumulation of resources outweighing the benefits of sexual reproduction (Vallejo-Marín *et al*., 2010).

Dispersal of fragments is common in aquatic systems where they are easily broken off and carried to new locations providing an additional means of dispersal (Eckert *et al*., 2016). Seagrass fragments can act as a dispersal mechanism and can facilitate long-distance dispersal (Kendrick *et al*., 2012; Berković *et al*., 2018). Vegetative fragments have the ability to disperse further than negative buoyant seeds such as in *H. nigricaulis* (Kendrick *et al*., 2012; Berković *et al*., 2014, 2018) and recent studies have demonstrated seagrass fragments may be able to disperse hundreds of kilometres (Berković *et al*., 2014; Stafford-Bell *et al*., 2015; Thomson *et al*., 2015; Smith *et al*., 2018). Matching clones at multiple sites in Port Phillip Bay adds further support that dispersal of fragments can occur at bay-wide scales (tens of kilometres). Fragments have significant dispersal advantages over seeds that need to be considered in an evolutionary context. In most circumstances, fragments should be able to outcompete seeds for space and resources during establishment (Eckert *et al*., 2016) and long-distance dispersal occurs at a much faster rate than via seeds (McMahon *et al*., 2014; Berković *et al*., 2018). Under circumstances where successful sexual reproduction is low due to unfavourable or stressful conditions, modular growth not only provides a means for population maintenance but also provides the opportunity for fragmentation and dispersal.

In marine systems, connectivity can be complex due to fluctuation in currents, winds and wave action, which act as the primary dispersal mechanisms (White *et al*., 2010; Geremew and Triest, 2019). Most studies rely on geographical over-water distance when comparing genetic structuring but often find weak to no relationship owing to the chaotic nature of large-scale and local conditions leading to stochastic recruitment (White *et al*., 2010; Sinclair *et al*., 2014*b*). Hydrodynamic models are being used increasingly to develop more realistic estimates of connectivity in aquatic systems (White *et al*., 2010; Geremew and Triest, 2019). We compared *F*_ST_ values with both over-water distance and total inflow (of propagules) and found propagule exchange via hydrodynamic models was better at predicating genetic connectivity, although variance accounted for in this model was still low (*R*^2^ = 0.15). Dispersal of the seagrass *Posidonia australis* across broad scales has shown similar patterns where there is a significant but weak relationship with oceanic dispersal models (Sinclair *et al*., 2016, 2018). Improvements in predicting genetic connectivity using hydrodynamic models as a proxy for distance between sites demonstrate the role of hydrodynamics dispersal of propagules in complex marine environments and the importance of long-distance dispersal (Sinclair *et al*., 2018). The overall low *R*^2^ values, however, may represent chaotic genetic patchiness recorded in marine environments from local stochasticity and recruitment bottlenecks (Becheler *et al*., 2010; Sinclair *et al*., 2014*b*, 2018). The complexity of the hydrodynamic model and life history traits included in the biophysical model will also play an important role in determining how accurately the model predicts the dispersal of propagules. Gaining a greater understanding of an organism’s life history traits such as settlement processes, propagule production and offspring survival will improve the accuracy of biophysical models and our understanding of connectivity in the marine environment.

Clonal life history traits have an advantage in habitats where the relative cost of sexual reproduction is high (Silvertown, 2008; Vallejo-Marín *et al*., 2010). We demonstrated that sites with large seed banks and seed production have high genotypic diversity relative to sites with low seed production. Sites with high genotypic diversity are also well connected genetically through propagule dispersal, allowing the import and export of novel genotypes. Isolated sites are located away from these suitable sites, and are often in more stressful environments where suitable habitats or resources are scarce. Consequently, investment in sexual reproduction is lower and modular growth may be more advantageous. Many modular organisms are key habitat providers (e.g. seagrass, coral). Understanding the role of sexual and asexual reproduction and dispersal in these organisms is important for predicting the response of these organisms to future global change and conservation.

## Supplementary Material

mcag008_Supplementary_Data

## Data Availability

Microsatellite data and biophysical modelling data are available in the Dryad data depository at https://doi.org/10.5061/dryad.cvdncjt4v.
