# Supplementary material for: Isolation leads to greater clonality and reduced seed production in a temperate seagrass
Source: Ann Bot. 2026 Jan 23;137(5):1404–15. doi: 10.1093/aob/mcag008 (PMC13197581; doi:10.1093/aob/mcag008)
Supplement: mcag008_Supplementary_Data [file mcag008_supplementary_data.docx]

**Supplemental Information for:**

**Isolation leads to more clonality, rather than seed production, in a temperate seagrass**

T. M. Smith, G. Bramwell, E. A. Treml, P. H. York, P. I. Macreadie, D. J. Ross, M. J. Keough, C. D. H. Sherman

**Table S1**. GPS locations of all site where genetic samples were collected in Port Phillip Bay, Victoria, Australia

| **Site** | **Latitude** | **Longitude** |
| --- | --- | --- |
| Ricketts Point | -37.993 | 145.029 |
| Avalon | -38.086 | 144.429 |
| Grand Scenic | -38.164 | 144.477 |
| Kirk Point | -38.037 | 144.556 |
| Point Henry | -38.143 | 144.416 |
| Pt Richards | -38.110 | 144.629 |
| Altona | -37.871 | 144.841 |
| Jawbone | -37.866 | 144.881 |
| Williamstown | -38.871 | 144.909 |
| Blairgowrie | -38.363 | 144.791 |
| Edwards Point | -38.194 | 144.711 |
| Mud Islands | -38.272 | 144.775 |
| Point Lonsdale | -38.275 | 144.630 |
| Rosebud | -38.355 | 144.901 |
| North Swan Bay | -38.202 | 144.684 |
| South Swan Bay | -38.270 | 144.634 |

**Table S2.** Summary microsatellite statistics for each loci.

| Loci | Allelic Richness (Ar) | Number of Alleles (Na) | Observed heterozygosity (H_O_) | Expected heterozygosity (H_E_) | Inbreeding coefficient (F_IS_) |
| --- | --- | --- | --- | --- | --- |
| ZosVic69 | 5.869 | 12 | 0.775 | 0.748 | -0.037 |
| ZosVic70b | 8.502 | 16 | 0.893 | 0.857 | -0.042 |
| ZosVic50 | 3.888 | 9 | 0.669 | 0.591 | -0.132 |
| ZosVic55 | 3.354 | 7 | 0.549 | 0.555 | 0.011 |
| ZosVic71 | 5.947 | 12 | 0.359 | 0.774 | 0.536 |
| ZosVic60 | 1.848 | 3 | 0.063 | 0.153 | 0.592 |
| ZosVic49 | 3.978 | 7 | 0.633 | 0.612 | -0.036 |
| ZosVic66 | 3.253 | 8 | 0.239 | 0.569 | 0.581 |
| ZosVic68 | 5.698 | 14 | 0.773 | 0.759 | -0.019 |

**Table S3.** Person correlations between reproductive, genetic and connectivity variables collected across all sites. R^2^ values followed by p values. Significant values in bold (p<0.05).

|  | R | Seed density | Spathe density | Na | He | Node-degrees |
| --- | --- | --- | --- | --- | --- | --- |
| Seed density | **0.647** |  |  |  |  |  |
|  | **0.009** |  |  |  |  |  |
| Spathe density | **0.593** | **0.614** |  |  |  |  |
|  | **0.019** | **0.015** |  |  |  |  |
| Na | 0.682 | 0.249 | **0.546** |  |  |  |
|  | 0.088 | 0.371 | **0.035** |  |  |  |
| He | 0.540 | 0.002 | -0.160 | 0.818 |  |  |
|  | 0.859 | 0.994 | 0.568 | 0.534 |  |  |
| Node-degrees | -0.025 | 0.001 | 0.239 | 0.398 | **0.354** |  |
|  | 0.928 | 0.997 | 0.391 | 0.142 | **0.195** |  |
| Total inflow | 0.486 | -0.025 | 0.350 | 0.242 | 0.076 | **0.592** |
|  | 0.066 | 0.930 | 0.201 | 0.385 | 0.787 | **0.020** |

Na = The mean number of alleles He = Expected heterozygosity; Node – degrees = degrees in the genetic network based on F_ST_; Total inflow = the relative number of propagules each site receives in the biophysical model.

**Table S4.** Pairwise Fst estimates for each seagrass population around Port Phillip Bay excluding the loci ZosVic66 and ZosVic 71.**
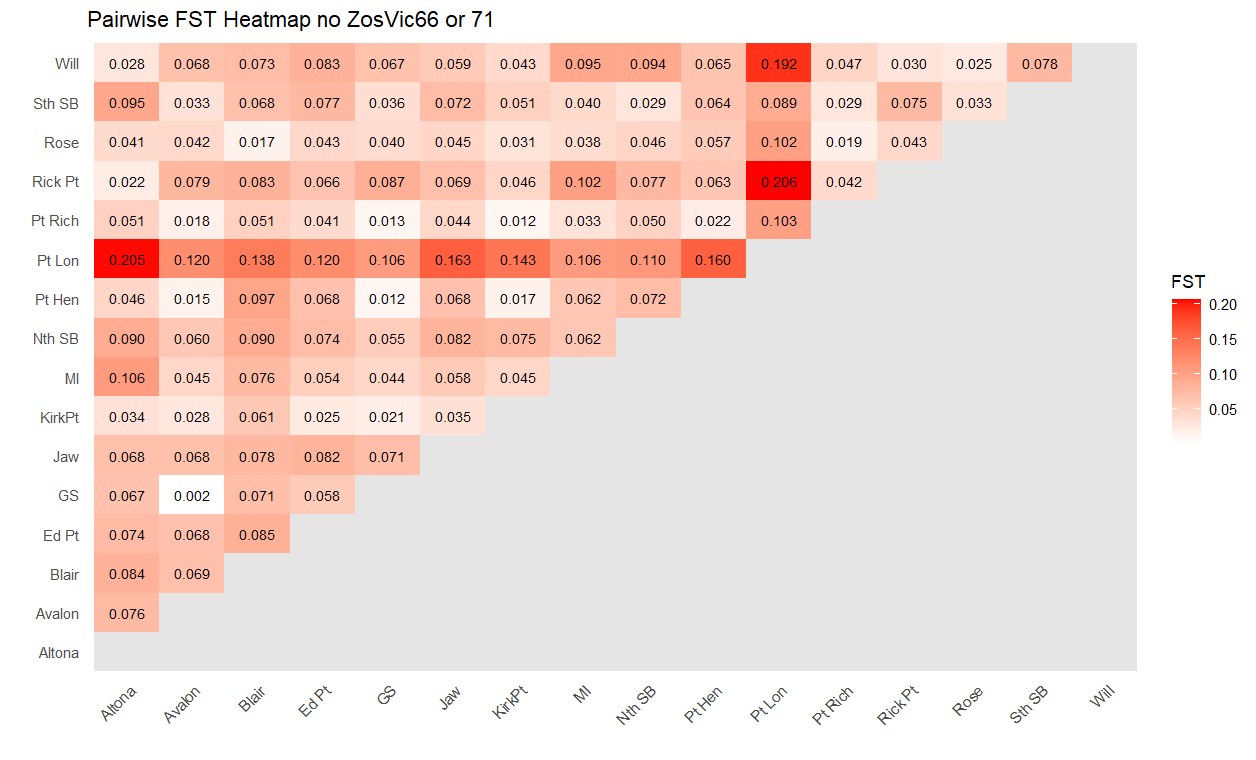
**


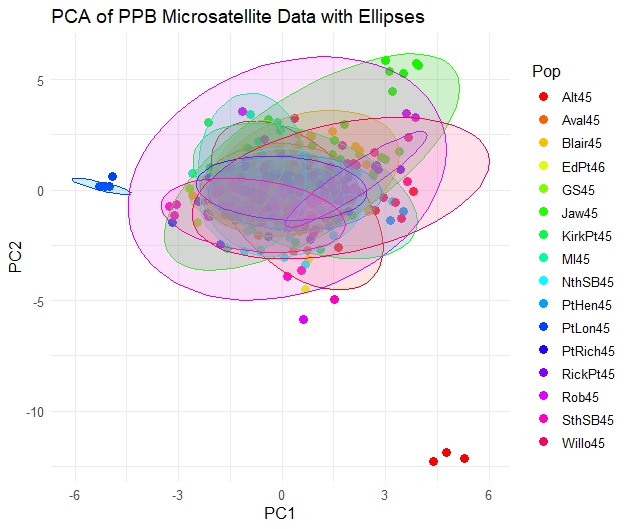


Figure S1. Principle Components analysis across 16 sampling sites in Port Phillip Bay.
